# Supplementary material for: Wound-induced syncytia outpace mononucleate neighbors during Drosophila wound repair
Source: eLife. 2026 Sep 22;13:RP92593. doi: 10.7554/eLife.92593 (PMC13597085; doi:10.7554/eLife.92593)
Supplement: Peer review history

# Editor report: eLife Assessment

**DOI:** 10.7554/eLife.92593.4.sa0

**Authors:** Sofia J Araújo

**Affiliations:** Universitat de Barcelona Spain

**Keywords:** Convincing, Important

This **important** work addresses a very relevant biological question: what is the cellular basis of wound healing? Using the *Drosophila* pupal notum as a model, the paper provides an elegant, thorough, descriptive characterization of syncytia-driven wound closure using state-of-the-art confocal live imaging of the pupal notum. The authors meticulously characterize the cell-cell fusion events during wound healing and inhibit cell fusion to show to that it is necessary to speed wound closure. This study provides **convincing** evidence that cell fusion allows actin resources at be partitioned to the leading edge.

# Referee report: Reviewer #1 (Public Review)

**DOI:** 10.7554/eLife.92593.4.sa1

[Editors' note: this version has been assessed by the Reviewing Editor without further input from the original reviewers.]

Summary:

This study aims to understand how cell fusion contributes to wound healing using a laser-induced injury in the notum epithelium of a developing fruit fly. The authors meticulously characterize the epithelial fusion events using a live imaging approach and report that syncytia arise by 'border breakdown' and 'cell shrinking'. The syncytial epithelial cells also appear to outcompete mononucleated cells and preferentially dissolve their tangential borders, which correlates with the accumulation of actin at the leading edge.

Strengths:

The strength of this study is the authors' live imaging approach to capture these dynamic fusion events that are a fundamental yet poorly understood biological process.

# Referee report: Reviewer #2 (Public Review)

**DOI:** 10.7554/eLife.92593.4.sa2

Summary:

Overall, this study provides a thorough description of the formation of syncytia following wounding of the proliferation-competent diploid epithelium of the pupal notum. While this phenomenon has already been described briefly for this particular tissue by the Galko lab in Wang et al 2015, the authors provide a much more detailed description and characterisation of the process providing some novel insights (radial versus tangential border breakdown, cell shrinkage, timings, syncytia outcompeting mononucleated cells, etc.).

Strengths:

This paper provides an elegant, thorough, descriptive characterisation of syncytia-driven wound closure using state-of-the-art confocal live imaging of the pupal notum. The authors show that laser-induced wounding of this diploid, proliferation-competent epithelium results in the formation of syncytia of various sizes in the first few cell rows around the wound edge, which progressively become bigger as healing proceeds. This results in ~50% of cells becoming part of these syncytia. The cell fusion events were convincingly demonstrated by showing the disappearance of p120ctnRFP and E-Cadherin-GFP from cell-cell borders as well as cytoplasmic GFP mixing of GFP-positive cells with a GFP-negative cell.

Apart from cell-cell fusion by border breakdown that mostly happens in the first 2h following wounding, the authors also found that at later stages of wound healing cell shrinkage following cytoplasmic mixing contributed to syncytia formation.

Next, the authors provided some convincing evidence that syncytia outcompete mononuclear cells for being positioned in the first cell row around the wound.

The authors then show that radial border breakdown occurs much less frequently than tangential border breakdown. They suggest that radial border breakdown reduces the requirement for cell-cell intercalations. They also hypothesise that tangential border breakdown might allow fused cells to share resources and provide more resources to be used near the wound edge, e.g. for actomyosin cable formation. To test this, the authors generate single-cell clones that overexpress Actin-GFP. They then show convincingly how a single Actin-GFP-positive cell in the second cell row fuses with one GFP-negative cell in the first cell row. The Actin-GFP signal then spreads in the fused cell and labels some previously unlabelled actin-rich structure near the wound edge which most likely is the actomyosin cable. This provides some evidence for resource sharing by cytoplasmic mixing following fusion.

# Referee report: Reviewer #3 (Public Review)

**DOI:** 10.7554/eLife.92593.4.sa3

In this revised manuscript, White et al. aimed to understand the wound-induced syncytia formation behavior in wound repair of *Drosophila melanogaster* pupal notum. For this purpose, the authors characterized two different types of adherens junctions' outcomes during syncytia formation around the wound region - border breakdown versus apical shrinking which appear to happen in different time points and for different time durations. The authors characterized cell-cell fusion events using cytoplasmic, junctional and nuclear markers. They determined that about half of the cells within 70 um radii from the wound undergo cell-cell fusion. They studied wound induction on the border between control epithelia and pnr domain suggesting that Atg1 is required for post-wound syncytia formation and wound closure. They showed that during wound closure syncytia gradually invade the wound leading edge mostly by radial fusion events. The data suggests that intercalation of cells from the leading edge slows down the wound closure process. They propose that cell fluidity of syncytial cells plays a role in wound closure speed. Finally, the authors showed that actin is concentrated to the front edge of syncytia located in the wound leading edge. The authors described some aspects of syncytia formation during wound closure using different approaches.

# Author comment: Author response

**DOI:** 10.7554/eLife.92593.4.sa4

**Authors:** James S White, Junmin Hua, Jasmine J Su, Kaden J Tro, Elizabeth M Ruark, M Shane Hutson, Andrea Page-McCaw

**Affiliations:** Vanderbilt University Nashville United States; Vanderbilt University Nashville United States; Vanderbilt University Nashville United States; University of California, Nashville Nashville United States; Vanderbilt University Nashville United States; Vanderbilt University Nashville United States; Vanderbilt University Nashville United States

The following is the authors’ response to the previous reviews

**Public Reviews:**

**Reviewer #1 (Public Review):**

Summary:

This study aims to understand how cell fusion contributes to wound healing using a laser-induced injury in the notum epithelium of a developing fruit fly. The authors meticulously characterize the epithelial fusion events using a live imaging approach and report that syncytia arise by 'border breakdown' and 'cell shrinking'. The syncytial epithelial cells also appear to outcompete mononucleated cells and preferentially dissolve their tangential borders, which correlates with the accumulation of actin at the leading edge.

Strengths:

The strength of this study is the authors' live imaging approach to capture these dynamic fusion events that are a fundamental yet poorly understood biological process.

Comments on revised version.

The manuscript overall is significantly improved and authors addressed majority of my concerns. The addition of the computational vertex model (Figure 7) as well as Atg1 RNAi (Figure 4) to inhibit cell fusion provide more mechanistic insight to their study. However, the analysis of Atg1 RNAi wound assay falls short as it does directly measure changes in syncytium frequency nor size to confirm that cell fusion is reduced. The authors should quantify the number of nuclei per syncytium over the 2hr wound healing period as performed for WT in Figure 1C. It would have been ideal if they could have also performed the Act-GFP spreading assay in WT and Atg1 RNAi strains to determine if Act-GFP movement is dependent on cell fusion as purposed. At the least, further quantification of Atg1 RNAi phenotype is warranted to support their conclusions.

In response to the reviewer's comment, we have repeated the analysis of Fig 1C and generated a new panel, Fig. 4C, which is directly comparable to the control and shows that syncytial size is dramatically reduced in the *Atg1* knockdown area. Unfortunately, we cannot perform the second analysis of actin-GFP spreading in the Atg1 knockdown cells because we need Gal4 for labeling individual cells and for knocking down Atg1, and we can't do both at the same time.

**Reviewer #2 (Public Review):**

Summary:

Overall, this study provides a thorough description of the formation of syncytia following wounding of the proliferation-competent diploid epithelium of the pupal notum. While this phenomenon has already been described briefly for this particular tissue by the Galko lab in Wang et al 2015, the authors provide a much more detailed description and characterisation of the process providing some novel insights (radial versus tangential border breakdown, cell shrinkage, timings, syncytia outcompeting mononucleated cells, etc.).

Strengths:

This paper provides an elegant, thorough, descriptive characterisation of syncytia-driven wound closure using state-of-the-art confocal live imaging of the pupal notum. The authors show that laser-induced wounding of this diploid, proliferation-competent epithelium results in the formation of syncytia of various sizes in the first few cell rows around the wound edge, which progressively become bigger as healing proceeds. This results in ~50% of cells becoming part of these syncytia. The cell fusion events were convincingly demonstrated by showing the disappearance of p120ctnRFP and E-Cadherin-GFP from cell-cell borders as well as cytoplasmic GFP mixing of GFP-positive cells with a GFP-negative cell.

Apart from cell-cell fusion by border breakdown that mostly happens in the first 2h following wounding, the authors also found that at later stages of wound healing cell shrinkage following cytoplasmic mixing contributed to syncytia formation.

Next, the authors provided some convincing evidence that syncytia outcompete mononuclear cells for being positioned in the first cell row around the wound.

The authors then show that radial border breakdown occurs much less frequently than tangential border breakdown. They suggest that radial border breakdown reduces the requirement for cell-cell intercalations. They also hypothesise that tangential border breakdown might allow fused cells to share resources and provide more resources to be used near the wound edge, e.g. for actomyosin cable formation. To test this, the authors generate single-cell clones that overexpress Actin-GFP. They then show convincingly how a single Actin-GFP-positive cell in the second cell row fuses with one GFP-negative cell in the first cell row. The Actin-GFP signal then spreads in the fused cell and labels some previously unlabelled actin-rich structure near the wound edge which most likely is the actomyosin cable. This provides some evidence for resource sharing by cytoplasmic mixing following fusion.

Comments on revised version:

The authors have extended their original manuscript by adding two key parts. First, they show a role of Atg1 in mediating cell fusion (Figure 4). Second, they provide additional evidence for a contribution of radial border fusions to wound closure through its effect on tissue fluidity and through computational modelling (Figure 7).

This new version of the manuscript is greatly improved and provides significant new insights into the role of syncytia in aiding wound repair. There are just a few minor, yet important, additions needed to back up Figure 4 which should not require new experiments.

Minor but important points:

The authors show a role of Atg1 in mediating syncytia formation in Figure 4. However, since the Pnr>+ side of the wound closes slower than the non-Pnr side (control side), a few additions to this figure would be important and should not require additional experiments.

(1) The authors should show, similar to the data shown in Figure 4D of the wound radius over time for control versus Pnr>Atg1RNAi, also the same type of data for control versus Pnr>+.

The data the reviewer requests is available in our bioRxiv manuscript, in Fig. 6B (Hua, Krystofiak, Pumford, Page-McCaw, and Hutson, https://doi.org/10.64898/2026.05.31.728998). These experiments were all done at the same time. As you can see, the difference in closure rate is quite subtle in control wounds.

(2) Since Pnr>+ also slows down wound healing, albeit to a lesser extent than Pnr>Atg1, the authors should also show an extra graph that provides evidence that Pnr>Atg1RNAi reduces syncytia formation more than Pnr>+ does. E.g. Two graphs could be added that show individual cell size at 4 or 5h post wounding for control versus Pnr>Atg1RNAi as well as for control versus Pnr>+ and also another graph with the same data but comparing cell size between Pnr>+ and Pnr>Atg1RNAi. Otherwise, if the expected minimum cell size for a syncytium is easy to estimate, a graph could be added that shows the percentage of cells that are above this threshold (e.g. above 100 square micron) for control versus Pnr>Atg1RNAi and control versus Pnr>+ and Pnr>+ versus Pnr>Atg1RNAi.

In response to this comment and the comment from reviewer 1, we have now added new Fig. 4C, which addresses the reviewer's question about the comparative frequency of fusion in *pnr>Atg1RNAi* and *pnr>+*. These graphs show that *Atg1* knockdown significantly reduces the size of syncytia.

**Reviewer #3 (Public Review):**

In this revised manuscript, White et al. aimed to understand the wound-induced syncytia formation behavior in wound repair of *Drosophila melanogaster* pupal notum. For this purpose, the authors characterized two different types of adherens junctions' outcomes during syncytia formation around the wound region - border breakdown versus apical shrinking which appear to happen in different time points and for different time durations. The authors characterized cell-cell fusion events using cytoplasmic, junctional and nuclear markers. They determined that about half of the cells within 70 um radii from the wound undergo cell-cell fusion. They studied wound induction on the border between control epithelia and pnr domain suggesting that Atg1 is required for post-wound syncytia formation and wound closure. They showed that during wound closure syncytia gradually invade the wound leading edge mostly by radial fusion events. The data suggests that intercalation of cells from the leading edge slows down the wound closure process. They propose that cell fluidity of syncytial cells plays a role in wound closure speed. Finally, the authors showed that actin is concentrated to the front edge of syncytia located in the wound leading edge. The authors described some aspects of syncytia formation during wound closure using different approaches. Some clarifications are needed as described below.

Major suggestions:

(1) Introduction, page 4. The examples of developmental syncytia formation of invertebrates and vertebrates are confusing. The authors may want to make the examples clear and add additional examples. Currently, readers may assume that *C. elegans* cell fusions occur only in the hypodermis - other structures can be mentioned like the vulva, pharyngeal muscles, glia, tail. In addition, the authors may want to add injury-induced fusions like the *C. elegans*' PLM and PVD neurons (Ghosh-Roy et al., 2010; Newman et al., 2015; Oren-Suissa et al., 2017).

We appreciate the suggestions and have included the additional examples of *C. elegans* vulva and PLM and PVD neurons. We are limiting ourselves to those because we don't want to focus too heavily on *C. elegans* examples, as that's not the direction this paper is heading.

(2) In cases where it is not clear whether fusion has occurred or whether mononucleated cells were ejected from the leading edge, membrane markers can be used. Page 6. Lines 96-99. The authors may want to use a membrane marker like RFP-PH driven by the epithelial cell promoter.

At this point in the manuscript, we are introducing syncytia and are not concerned yet with their origin.

(3) Pages 8-10. The authors may want to clearly explain that apical junctions shrinking is a post fusion event. That the apical shrinking is caused by the expansion of fusion pores and the migration of apical junctions towards the basolateral domain. This is something that was clearly shown during physiological epidermal cell-cell fusion in *C. elegans* by Mohler et al., 1998 and 2002. A cartoon showing the process of cell-cell fusion, pore expansion and apical junction dynamics would make the manuscript much clearer.

Apical shrinking cannot be caused by the "migration of apical junctions towards the basolateral domain" because that is not what we observed -- rather, we observed labeled adherens junctions remaining at the apical surface while the area they enclose becomes smaller (shrinks). Further, despite close reading of the Mohler papers, it is not clear how similar the apical shrinking events of this manuscript are to the fusion events described there. Finally, we do not want to include a schematic describing this process because that would suggest certainty that we do not have. Unlike in *C. elegans* development, wound-induced cell fusion is stochastic, not stereotyped; with cells that display apical shrinking, the fusion partner of a labeled cell is difficult to identify because it is often not a neighboring cell. These factors make it difficult to describe this process in detail, but we have sufficient data to conclude that these are indeed cell fusion events.

(4) Page 9. Line 170. "...as these cells represent fusion initiation events (fusion pore) but were unable to productively stabilize and expand the site of fusion and so returned to the diploid state." The authors may want to make clear that this is an assumption that needs to be tested. Live imaging using a membrane marker may resolve whether a reversible fusion pore was generated.

Thank you for the suggestion; we have updated this text to make it clear that this is an interpretation.

(5) Page 11. It is not clear whether Atg1 is directly required for cell fusion, or that autophagy is required for efficient cell fusion or both Atg1 and autophagy participate in the fusion process.

Our data show that *Atg1* is required for cell fusion. The work that inspired this experiment, Kakanj et al 2022, concluded from their more comprehensive studies that the process of autophagy was required. We have clarified the text.

(6) Page 12. Line 235. "Indeed, we observed that several hours after wounding, the entire leading edge was occupied by syncytia." This observation is based only on the adherens junction marker. Can they test basal cell membrane marker? Is it possible that the mononucleate cell in the leading edge is under the two syncytia?

Unfortunately, there are not good basal markers -- the recently reported basal spot markers also label adherens junctions. Nonetheless, we are confident that the mononuclear cell is not under the syncytia because we image Z-stacks and thus can detect cell overlap.

**Recommendations for the authors:**

**Reviewer #3 (Recommendations For The Authors):**

Minor suggestions:

(1) Figure 1. The authors may want to add an image immediately after laser ablation of the actual wound and the area around the wound. Add an arrow to mark the wound.

With this wounding modality, the extent of the wound is unclear for ~30 min. As we reported in O'Connor et al, PLoS One, 2021, there is a gradient of damage emanating out from the center of the wound, and cells with greater amounts of damage die while those with less damage repair and survive. Immediately after laser ablation, very little visible damage is evident by 120ctn-RFP and Histone-GFP (the markers in Fig. 1) until the cells die and the surrounding cells respond.

(2) Page 6. Line 86. "A mitotic tissue utilizes cell-cell fusions during wound repair." replace "during wound repair" with "after wound induction" since in this section the authors do not show that this process is part of wound repair.

Thank you for the suggestion - we reworded this heading to remove "wound repair".

(3) Page 6. Line 92. The authors may want to be consistent with the terms used in the text and in the figure - His2GFP in the text versus Histone GFP in the figures.

Thank you for the suggestion, we have revised for consistency.

(4) Figure 1 - supplement figure 1D. The "v" of Div panel moved below D.

Thank you, we have corrected it.

(5) Figure 1 - supplement figure 1G. add "i" to second Gii to make it Giii.

Thank you, we have corrected it.

(6) Page 24. Figure 1H legend. 3 or 4 wounds?

Thank you for catching this error - 4 wounds.

(7) Page 7. Line 124. "GFP mixing always preceded border breakdowns (n=11)" instead of "always" use "in all observed cases".

We have made this change.

(8) Figure 2. Switch the writing "Apical Shrinking: Nuclear Transfer" since apical shrinking represented in panel 2A and Nuclear Transfer in panel 2B. If this description applies only to panel 2B, make it clear.

We consider this heading to apply to panels A and B together (as they show the same sample, just different channels).

(9) Figure 2C. Is ActinGFP a cytoplasmic GFP driven by actin promoter or Actin-bound GFP? Cytoplasmic GFP versus membrane-cortex GFP?

It is a transgene expressing an actin-GFP fusion protein, as noted in the key reagents table and discussed in Fig. 8. We corrected the manuscript to ensure it is always referred to now as Actin-GFP in the text, figures, and legends.

(10) Video 3 - Impressive movie!

Thank you!

(11) Page 9. Line 155. "In both these cells, as the cell lost its basal volume, cytoplasm moved laterally to join the neighboring syncytia." It seems that the apical shrinking cells' cytoplasm joined the neighboring syncytia even before.

Because both indicated cells (yellow and white arrows) and the neighboring syncytium are all labeled with GFP, it is not possible to determine precisely when the cells' cytoplasm joined the syncytium.

(12) Page 9. Line 158. "...but fusions associated with apical shrinking occurred later and were more numerous." Did the fusion occur later or the apical shrinking itself as was mentioned before and shown in Figure 2F?

We have changed the wording, as for many apical shrinking events we cannot tell exactly when the fusions were initiated.

(13) Page 25. Figure 3A legend. What is the meaning of morphological fusion? Border breakdown and apical shrinking? The authors may want to define it.

We have defined it now in the legend.

(14) Page 26. Figure 3B-C legend. "Panel C shows that apical shrinking fusion and border-breakdown fusion occur at similar distances from the wound." It seems that fusion by apical shrinking mostly occurs within 60-70 um from wound center and fusion by breakdown occurs equally at all distances up to 80 um.

We don't disagree with your comment, but we feel the dataset is too small to make such a statement. The data is presented so the interested reader can make their own conclusion.

(15) Page 9. Line 165. "...but infrequently (n=3) with GFP mixing and no subsequent cell fusion..." Does this mean that there were GFP mixing without border breakdown or apical shrinking?

Yes, that is correct. We assume that in this case a fusion pore opened and then closed again. We have added a phrase to clarify.

(16) Page 9. Line 175. "...the spatial distribution of fusing cells that shrank vs. lost borders was similar (compare Figures 1G and 2E)." Even though the visual comparison suggests similar spatial distribution, the carefully quantified distribution in figure 3C suggests more fusion by shrinkage at 60-70 um from wound center of the 5 tested wounds.

As we noted to comment 14, we feel the data set is too small to make such a statement. The data is presented so the interested reader can make their own conclusion.

(17) Figure 3. The shown pies sum the results from 5 wounds. It would be interesting to add a graph comparing the percentage of fused and persisted cells per wound to see the variability, if exists.

Unfortunately, the number of fused/persisting cells in each wound is greatly affected by the heat-shock conditions that generate the labeled clones; even the ratio of these fates would be heavily influenced by noise because the numbers are small in each animal. Further, the frequency of fusion is determined by the wound size as shown in Fig. 1. Because of these variables, such data could be easily misinterpreted.

(18) Figure 3 - figure supplement 1D. Even though it was mentioned that the duration of some border breakdown is finished within minutes it is worth comparing it with shrinking duration on one graph.

Unlike apical shrinking, it is difficult to identify exactly when border breakdown concludes, so this data is difficult to compare. We have provided several examples of border breakdown in the manuscript that give an overview of the process.

(19) Video 1 is not mentioned in the main text.

Thank you for catching that omission. We now refer to it in the first paragraph of the results.

(20) Figure 4B. The difference between the treated group and the control group is unclear. Add arrows.

We have added some arrows to Fig. 4B.

(21) Figure 4C. For consistency use percentage for both border breakdown and shrinking cells.

In response to the reviewer's comment, we now provide the consistent metric of number of lost borders and number of shrinking cells.

(22) Page 11. Did the authors try other wound types (e.g. mechanical/chemical wounds)? May other wound causes besides laser ablation result in different response? This may help to answer whether there is a causation between syncytia formation and speed wound closure.

There are reports of puncture and pinch wounds inducing cell fusion. Perhaps the reviewer is suggesting that we might be able to identify a wounding method that does not induce cell fusion and then compare the rate of wound closure. However, another type of wound would probably inflict different amounts of cell damage and so would be hard to compare. Overall, we think the half-and-half system of comparing responses on the two sides of the wound is the best, most controlled comparison.

(23) Figure 4F-G. It was mentioned that there is less syncytia formation in Atg KD cells, however the difference in cell area between control, WT and Atg KD is not obvious. The authors may want to mark the dots that represent syncytia to distinguish them from mononucleated cells.

The point we are trying to make (now Fig. 4G-H) is that cell area is related to distance moved, regardless of how cell area is determined. We do not have the ability to count nuclei in the control sides (nuclei are labeled only on the *pnr* side), and further, we have reported separately (White et al, 2024) that there is a limited amount of endocycling in these cells, which should also increase area.

(24) Figure 5G. y axis. The authors may want to change "small cells" to "mononucleate cells".

We changed it to "unfused cells" which is the term we used in the legend. In these wounds we were unable to visualize nuclei.

(25) Page 12. Line 243. (Figure 5D,G) instead (Figure 5D).

We changed it to read (Figure 5D,G).

(26) Page 12-13. Lines 241-246. The description of "mononuclear cells removed" and "syncytia outcompete unfused cells" may be clearer if explained here as mononuclear cells joining the syncytium by cell-cell fusion.

Here we are describing a different phenomenon - not that fusion is removing all the smaller cells but rather that the syncytia are faster/better/more effective at wound closure than the smaller cells. This is illustrated in Fig. 5Cii-Ciii.

(27) Page 13. Line 261. "Thus, there were about five-fold more tangential borders lost to fusion than radial" Is this conclusion also true when analyzing each wound individually?

This is a reproducible finding, that there is more fusion across tangential borders than across radial borders. The ratio of tangential-border loss: radial-border loss for each wound is as follows:

wound 1, 63:12

wound 2, 39: 11

wound 3, 44:8

wound 4, 50:8

(28) Page 31. Figure 6 - figure supplement 1 legend, Line 652. Make "B" bold.

Done.

(29) Page 14. Lines 270-275. If there is an advantage to radial fusion versus cell intercalation for wound closure speed, how do the authors explain that the percentage of radial fusion is lower than the percentage of intercalation? (Figure 6D) How does the wound affect the molecular level (fusogen expression?) of the surrounding cells?

We expect that radial fusion specifically reduces the need for intercalation at the leading edge, as shown in Fig. 6C. Both would speed closure, however, as any increase in cell area will allow more efficient redistribution of resources such as actin and will also reduce the total number of junctions needing to be remodeled as the wound closes. Since we don't know the fusogen, we can't say how the wound affects its distribution.

(30) Page 14. It is not clear where the experimental data ends and the model starts. For example, in line 276, it would be clearer to describe the "tissue fluidity as measured" or is it more precise to write instead "as estimated/calculated". The fusion between observations and model is confusing and maybe this should be unfused.

This text, referring to the analysis in Fig. 7A, B, is not a computational model but rather a quantitative analysis of tissue fluidity as measured by a pre-existing metric, the shape index. This is experimental data. The computational model begins in the next paragraph, accompanying Fig. 7C, D. We edited the language slightly in this paragraph to clarify.

(31) Figure 8 versus Figure 2C. Actin-bound GFP versus cytoplasmic GFP? Both mentioned as Actin GFP. Make it clear.

They are indeed the same thing, actin protein fused to GFP, as described in the text and legend, and they are labeled identically.

(32) Figure 8Biii, Div. Nice presentation of signal distribution between the cells.

Thank you!

(33) Page 30. Figure 8G legend. Lines 623-628. Is the shown mean profile plot based on specific images shown in Fi and Fv or just the cells represented there? Since Fi is a single z slice and Fv is maximum intensity projection which are not comparable.

In response to the reviewer's question, we reanalyzed the image. Fig. 8G compares Z-projections.

(34) Page 15. Line 303-304. "Tangential border fusions allow resources from distant cells to be mobilized to the wound edge." Does not this leading-edge actin localization happen in radial fusions close to the region of the wound?

Fusions along radial borders, as shown in the top panel of Fig. 6A, would not offer the opportunity to move actin from distant cells to cells nearer to the wound.

(35) Page 15. Did the authors test any predictions from the simulations of the model experimentally?

This isn’t so much a predictive model as an exploratory model that addresses one question: is it plausible that the presence of syncytia can speed closure by reducing the need for intercalations, even if the syncytia have no other special properties. The only prediction would be that inhibiting fusion would slow wound closure.

(36) Page 17. It would be interesting to discuss the following questions: (A) Is autophagy required for fusion. (B) Is Atg1 required for epithelial cell fusion? (C) Is autophagy required for wound repair? Are any of the combinations correct (A&B, A&C, B&C, A&B&C)

The role of autophagy in wound-induced cell fusion was thoroughly explored in the 2022 EMBO J paper from Maria Leptin's lab, "Autophagy-mediated plasma membrane removal promotes the formation of epithelial syncytia" by Kakanj et al. We merely knockdown a gene they discovered to be important for wound-induced epithelial fusion, *Atg1*, as one means of investigating how syncytia contribute to wound closure. Our results don't add to their findings, and the role of autophagy is not what we want to focus on in our Discussion.

(37) Page 19. Line 381-384. "If N represents the number of cells that fused, our results suggests that syncytia can apply up to N times more actin to the leading edge; considering that we observed syncytia with dozens of nuclei, this could represent a significant enhancement of actin at the leading edge. Increased actin might explain the ability of syncytia to outcompete diploid cells at the leading edge." To enhance this suggestion, the authors may want to compare actin signal in the leading edge of different size syncytia.

We thought a lot about this experiment because reviewer 2 asked for it in the previous round of review, but as we said then, we can imagine too many caveats to the interpretation to make it worthwhile.

(38) Page 22. Line 447-448. "...fusion would act the fastest after wounding because there is no need for DNA replication." There may be a potential need for protein (fusogen) synthesis.

The timing of fusion, which we report here begins within 10 minutes after wounding, suggests that if there is a fusogen, it is already present in the cells before wounding.

(39) Page 34. Line 716. Add "C" to "29{degree sign}".

Done.

(40) Page 38. "Wound closure analysis" part. Can the wound closure be visualized using brightfield?

The scar also impedes imaging through bright-field microscopy.
